# Representation of Spatial Variability of the Water Fluxes over the Congo Basin Region

**DOI:** 10.3390/s22010084

**Published:** 2021-12-23

**Authors:** Marc De Benedetti, G. W. K. Moore, Xiaoyong Xu

**Affiliations:** 1 Department of Physics, University of Toronto, Toronto, ON M5S 1A7, Canada; 2 Department of Chemical and Physical Sciences, University of Toronto Mississauga, Mississauga, ON L5L 1C6, Canada; xiaoyong.xu@utoronto.ca

**Keywords:** precipitation, evapotranspiration, model resolution, spatial variability, satellite precipitation

## Abstract

The Congo Basin, being one of the major basins in the tropics, is important to the global climate, yet its hydrology is perhaps the least understood. Although various reanalysis/analysis datasets have been used to improve our understanding of the basin’s hydroclimate, they have been historically difficult to validate due to sparse in situ measurements. This study analyzes the impact of model resolution on the spatial variability of the Basin’s hydroclimate using the Decorrelation Length Scale (DLCS) technique, as it is not subject to uniform model bias. The spatial variability within the precipitation (P), evaporation/evapotranspiration (E), and precipitation-minus-evaporation (P-E) fields were investigated across four spatial resolutions using reanalysis/analysis datasets from the ECMWF ranging from 9–75 km. Results show that the representation of P and P-E fields over the Basin and the equatorial Atlantic Ocean are sensitive to model resolution, as the spatial patterns of their DCLS results are resolution-dependent. However, the resolution-independent features are predominantly found in the E field. Furthermore, the P field is the dominant source of spatial variability of P-E, occurring over the land and the equatorial Atlantic Ocean, while over the Southern Atlantic, P-E is mainly governed by the E field, with both showing weak spatial variability.

## 1. Introduction

Central Africa has been identified as one of the regions where the effects of land surface conditions on regional climate and dynamics are most pronounced [1]. The Congo Basin in Central Africa covers an area of approximately 3.7 million square kilometers and contains the Congo River (the world’s second largest river in terms of discharge volume) and a large amount of tropical forests and wetlands, thus having an important impact on global hydrology and climate [2]. For example, deforestation in the Congo Basin region is leading to a decrease in surface evapotranspiration, which affects energy partitioning between sensible and latent heat surface fluxes and thereby the global climate through atmospheric teleconnections [3,4,5]. However, the Congo Basin has been one of the least studied tropical regions [2].

Given the global importance of the Congo Basin [2,4,5,6,7], there is a need to better understand the hydrological cycle behavior over this region. A key constituent of the hydrological cycle is the net water flux into the surface [8], calculated by precipitation minus evaporation or evapotranspiration (commonly referred to as P-E), which regulates land surface runoff and subsurface flow. In the long-term mean, changes in the surface water storage are negligible, and P-E equals runoff [9,10,11,12]. Investigation of P-E is an important aspect for revealing how a changing climate affects the hydrological cycle [8,13]. The long-term rain gauge measurements are sparse over the entire Congo Basin, which has hindered the identification of the long-term trend in basin-averaged precipitation using in situ data [14]. The northern and eastern portions of the Congo Basin were observed to be getting drier during the 1950s–1990s [15,16]. Recently, the satellite and model (reanalysis) products identified a long-term decline in April–June precipitation over the Congo Basin [7,17,18,19]. The Congo Basin has also experienced an increase in the areal extent and intensity of thunderstorms from April–June [20] and an increased dry season (June–August) length over the past few decades [21]. However, the different reanalysis datasets typically showed clear discrepancies in the magnitude and spatial pattern of precipitation trends over the Congo Basin [19,21]. It is expected that the model resolution has an important control on the spatial distribution of the Congo Basin precipitation trends derived from the model/reanalysis products [22,23].

Accurate estimation of the spatial pattern of the P-E field is crucial for understanding the response of P-E to global warming [8]. Investigating and visualizing the spatial variability of a single geophysical field is challenging enough; however, P-E is the difference between two fields, and thus it is not clear how the underlying spatial variability of its constituents is reflected in its spatial variability. Part of this difficulty stems from biases in the two constituent fields and their correlation, resulting in complex uncertainties in the P-E field [12,24]. The method used here is to study the spatial variability of both the precipitation (P) and evaporation (E) fields separately and then compare their results to that of the P-E field.

The Congo Basin is difficult and somewhat dangerous to access, and as such, there is a scarcity of in situ hydrometeorological measurements [2,25,26]. This poses an obstacle to investigating the spatial variability of the hydro-climatological fields over the whole Congo Basin with in situ data. To this end, attempts have been made to use other types of data, for example, characterizing the precipitation behavior of the region with satellite measurements and/or coarse-scale gridded gauge analysis [7,26,27,28,29].

**Figure 1:**
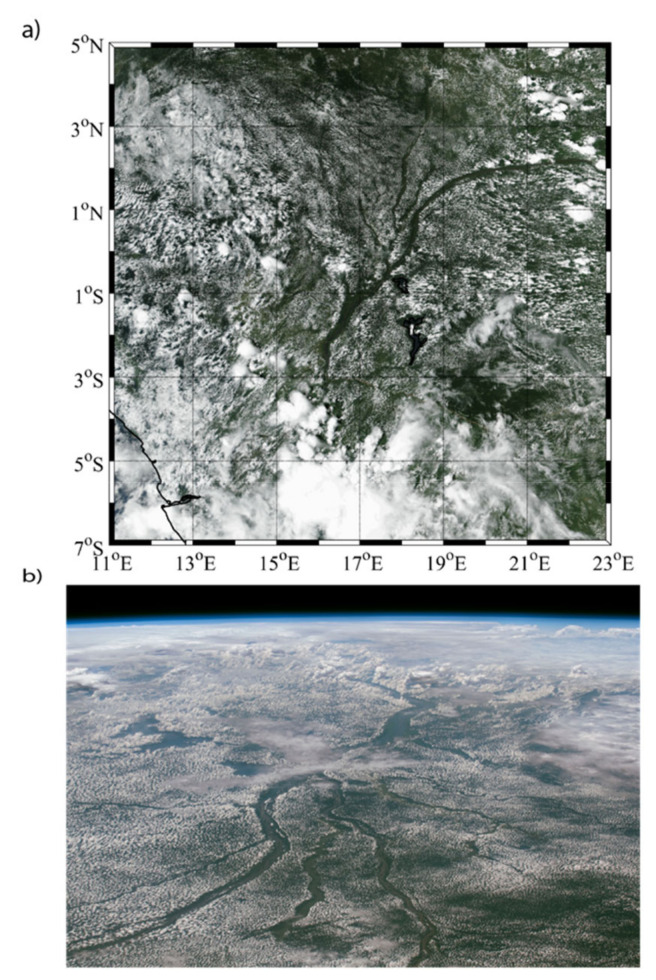
(**a**) True color MODIS satellite image of the Congo Basin centered over the Congo River on 5 November 2018 taken from NASA Worldview. (**b**) Shows the Congo River from the International Space Station (ISS), centered at the same location as the above MODIS image. NASA photo ID: ISS057-E-58903.

Model data-based studies play an important role in examining the hydrology and water fluxes over the Congo Basin and Western Equatorial Africa [11,30,31,32,33]. However, the region is subject to modeling challenges for both precipitation and evaporation. The representation of topography within models is a critical factor, influencing the modeled precipitation since precipitation cloud formation (e.g., meso-scale convective systems) in the area is controlled largely by orographic effects, the movement of the intertropical convergence zone, and large-scale circulation [27,31,34]. To illustrate the complexity within the cloud field, Figure 1 shows a MODIS true color image and a photo from the International Space Station for the Congo Basin (centered over the Congo River) on 5 November 2018. It can be seen in both images that the cloud cover is broken up into smaller cells over the land, and free of clouds over the Congo River, presumably as a result of its cooler surface. Precipitation from such clouds will occur on scales ~10 km, thus motivating the need to determine how model resolution impacts the representation of the precipitation field.

Land surface evaporation/evapotranspiration processes are typically affected by atmospheric conditions, such as radiation, air temperature, humidity, and wind, as well as land surface variations, such as those in vegetation cover, leaf area index, soil type, soil water content, and topography [35,36,37]. Modeling evaporation over the Congo Basin area is also a challenge due to the spatially varying vegetation cover, topography, soil type, and seasonal precipitation [2,35,38]. The evaporation data discussed in the present work are all from the European Centre for Medium-Range Weather Forecasts’ (ECMWF) Integrated Forecasting System (IFS), which used a land-surface scheme (TESSEL in ERA-Interim; HTESSEL in ERA5 and ECOA) to produce evaporation [39,40]. IFS-derived evaporation is sensitive to vegetation type and leaf area index [40].

**Figure 2:**
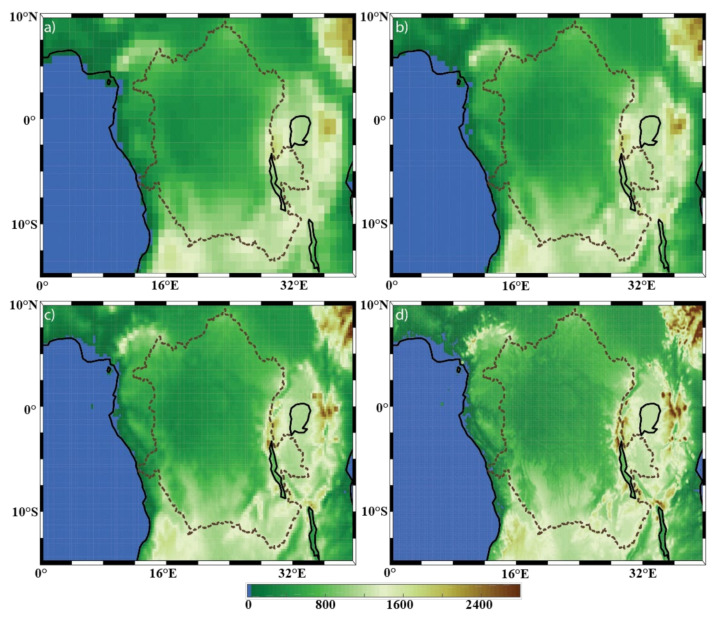
Topography (measured in meters, m) of the DRC region with the Congo Basin outlined with the dotted lines, derived from (**a**) ERA-I, (**b**) eERA5, (**c**) ERA5, and (**d**) ECOA, respectively.

There is a substantial spatial variability in surface conditions, such as topography (e.g., Figure 2, showing the topography of the region for each of the four model resolutions) and vegetation type (e.g., Supplementary Figures S1 and S2) over the Congo Basin and surrounding areas. Model resolution should impact the representation of these factors. Therefore, there is concern that coarse-resolution model (reanalysis) data, due to the possible underrepresentation of these surface variations, may not sufficiently capture the spatial variability pertaining to the precipitation, evaporation, and P-E fields in the region. However, this concern has not yet been clearly addressed. The present study is intended to fill this research gap. In this work, the decorrelation length scale (DCLS) [41], a novel metric that allows visualization of spatial variability in any geophysical field, will be used to investigate the spatial variability of the three fields (precipitation, evaporation, and P-E) and the impact of model resolution on them. As noted in [42], the DCLS is able to reveal spatial variability at a grid point-scale, whereas other methods (e.g., the power spectrum method used in [43]) usually provide only area averaged information.

The research objectives of this work include (i) characterization of spatial variability of water fluxes (P, E, and P-E) over the Congo Basin using model-based reanalysis data, and (ii) investigation of how their spatial variability varied with model resolution. The ultimate goal is to quantify the representation of spatial variability of the three water fluxes in model-based reanalysis products across different resolutions, providing critical guidance for the use of these data in hydro-climatological analysis and/or hydrological modeling activities.

## 2. Materials and Methods

This study aims to characterize both spatial variability and the impact of horizontal resolution on the representation of the Congo Basin’s hydrological cycle. Spatial variability is investigated using DCLS analysis, and the impact of model resolution on representation is examined using a variable-resolution model suite ranging from 75 km to 9 km.

### 2.1. Datasets

The precipitation and evaporation fields were taken from four datasets, including the ~75-km ERA-Interim (ERA-I) reanalysis [44], ~62-km ERA5 ensemble (eERA5) reanalysis [45], ~31-km ERA5 reanalysis [45], and the ECMWF’s ~9 km operational analysis (ECOA) [46]. For the eERA5, we use the single unperturbed ensemble member rather than the ensemble mean, as the latter proved to act as a smoother and may underrepresent the spatial variability present in individual ensemble members. The four datasets have a common lineage of the ECMWF’s Integrated Forecast System (IFS), which ensures that their DCLS results mainly reflect the variability in horizontal resolution rather than differences in model physics.

The four datasets have different output time steps (6 h for ERA-I and ECOA, 3 h for eERA5, and 1 h for ERA5). Given that the focus of this investigation was the impact of spatial resolution, all data are sampled to a 6-h time-step for the DCLS analysis. The datasets also have different temporal coverage periods. In this study, all DCLS analysis results are based upon a 2-year period (January 2016 to December 2017), which is the longest common time coverage (at the time of this analysis) for all four datasets.

### 2.2. The Decorrelation Length Scale (DCLS) Analysis Method

**Figure 3:**
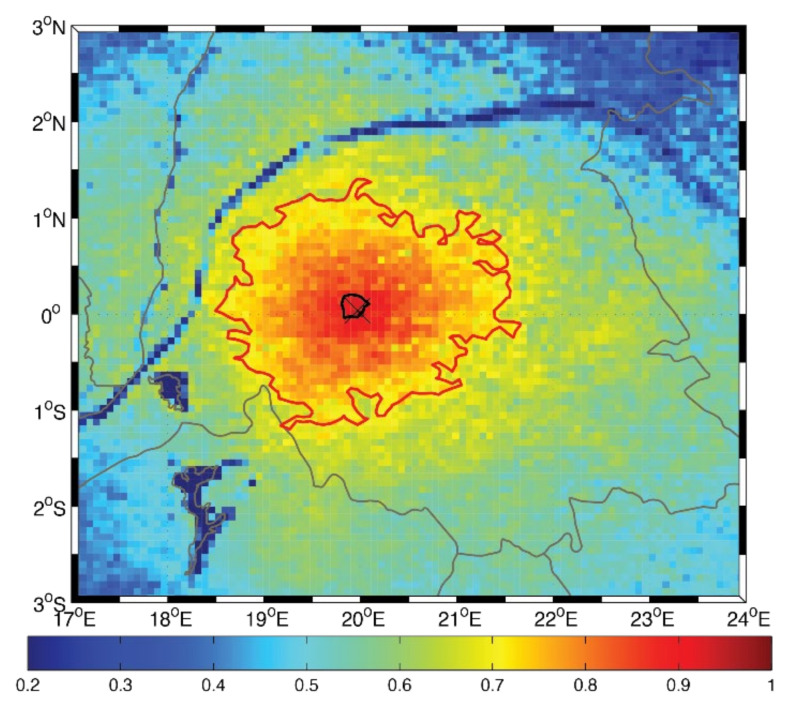
Example calculation of the DCLS value at location (20° E, 0° N). The Congo Basin’s boundary can be seen in gray. The background color represents the correlation coefficient matrix, and the black and red contours represent the 0.9 and 0.7 contour levels, respectively. The associated DCLS for the black 0.9 contour level is 16 km, whereas the DCLS for the red 0.7 contour is 140 km.

The DCLS analysis is a diagnostic tool used to characterize and visualize spatial variability by computing the decorrelation length scale (DCLS) value at each grid point in a gridded dataset, thus providing granular data on the spatial variability. As described in [41], the analysis mainly consists of the following steps. The first step is to compute the correlation coefficient for the time series of a field (e.g., precipitation, evaporation, or P-E) between a selected, fixed grid point and all other grid points in the dataset to produce a correlation coefficient matrix (e.g., the background shading of Figure 3). In this study, the correlation coefficients were computed using the 6-hour data for the period from 1 January 2016 to 31 December 2017. It was shown in [42] that the spatial patterns of the DCLS analysis remained unchanged for any period of time longer than 2 years. Second, the correlation coefficient matrix is contoured using a prescribed contour level (e.g., r = 0.9 for this study). Third, the DCLS value for the selected grid point is defined as the average distance between the fixed grid point and the aforementioned contour of the correlation coefficient matrix. This process is repeated until DCLS values are obtained for all grid points in the dataset. It should be noted, however, that since this analysis is based on correlation, geophysical fields that exhibit sharp changes in value (such as precipitation) might need to be diurnally averaged to reduce temporal noise, whereas fields that typically have continuous values (such as wind or evaporation) will not need to be averaged/smoothed. More detail on this calculation was provided in [41].

### 2.3. Interpreting Results of the DCLS Analysis

Since the DCLS value represents the average radius of the prescribed contour, its specific magnitude depends upon the prescribed contour level. This magnitude dependence can be seen in Figure 3, where the black contour represents the 0.9 contour level (DCLS = 16 km) and the red contours represent the 0.7 contour level (DCLS = 140 km). Instead, the relative magnitudes in DCLS values are what indicate regions of variability. If a region has a local minimum in DCLS values, this implies that the region has a higher degree of variability compared to surrounding regions. These spatial patterns can provide insight into the source of the variability (discussed in the Results section). As can be seen in Supplementary Figure S3, there is a strong linear relationship between DCLS values at the 0.7 and 0.9 thresholds, indicating that the choice of a 0.9 threshold will mainly change the magnitudes (this idea is discussed further in Section 3.3). However, it should be noted that the different fields produce different slopes. This is a measure of how quickly the magnitudes changes as the contour level changes. Fields with smaller slope values are less sensitive to the choice of DCLS value, whereas fields with larger slope values may indicate higher sensitivity—suggesting the possibility that a larger threshold is needed to capture the variability as it may occur on smaller spatial scales.

An added benefit of the DCLS analysis is that it is not sensitive to systematic model bias. The DCLS value is calculated using the correlation coefficient matrix, which is not strongly influenced by systematic biases in the data. The DCLS analysis can also be used to assess the impact of model resolution on the representation of geophysical fields by investigating how the spatial patterns in the DCLS analysis changes across different resolutions. The presence of resolution-dependent DCLS spatial patterns may indicate that the coarser-resolution models underrepresent the spatial variability of the given field.

For the purposes of this initial paper, the focus will be on characterizing the spatial variability of annual mean precipitation and evaporation fields over the Congo Basin. As noted in [2], there is a seasonal migration of the region of maximum precipitation from the north during the July–October period to the south during the December–March period that is associated with seasonality in the tropical rainbelt [30]. In subsequent work, seasonality in the DCLS of the hydroclimate variables will be examined.

### 2.4. Validation of the Model Datasets

It is acknowledged that the detailed validation (especially against in situ observations) of the model datasets has merit. However, it is not practical to use in situ measurements to evaluate the four model datasets over the Congo Basin due to the well-known scarcity of in situ hydrometeorological observations for the region [2,25,26]. As such, satellite products are the key data sources that can be used as a reference to validate the model datasets over the Congo Basin. It has been demonstrated that the TAMSAT satellite precipitation product performs very well over Africa [47,48,49]. In this study, the precipitation (P) fields from the four model datasets are validated using the TAMSAT-3 rainfall data [47,48,49]. Supplementary Figure S4 compares the precipitation from each of the four model datasets used against the TAMSAT-3 satellite-based precipitation product. The spatial distribution of TAMSAT-3 precipitation can be seen in Supplementary Figure S4a and visually matches that of the four model datasets (see Section 3.1). Furthermore, the subsequent four panels (b–e) show scatter plots between the model precipitation and TAMSAT-3 precipitation. Note that each of the five datasets was mapped to the latitude/longitude grid from ERA-I, as it has the lowest resolution. These scatter plots show a strong linear relationship with high R^2^ values and slopes close to 1. It can be seen that ERA-I (Supplementary Figure S4b) is the only dataset that produces a slope larger than 1, further supporting the known positive bias in precipitation values within ERA-I over the Congo Basin [25,32]. Note that a more quantitative validation analysis for the model datasets using absolute error measures (e.g., bias and RMSE) is beyond the scope of the present study because the absolute error measures are not closely related to the DCLS analysis of P, E, and P-E in the model datasets. The DCLS analysis is independent of the systematic errors in the model datasets.

## 3. Results

As mentioned in the Introduction, the precipitation and evaporation fields are studied individually before considering P-E. The analysis will start with analyzing the climatology of the precipitation, evaporation, and P-E fields, which will provide some insight into the impact of model resolution on the mean hydrological cycle, and then investigate the spatial variability of the three fields using the DCLS technique.

### 3.1. Mean Annual Fields

**Figure 4:**
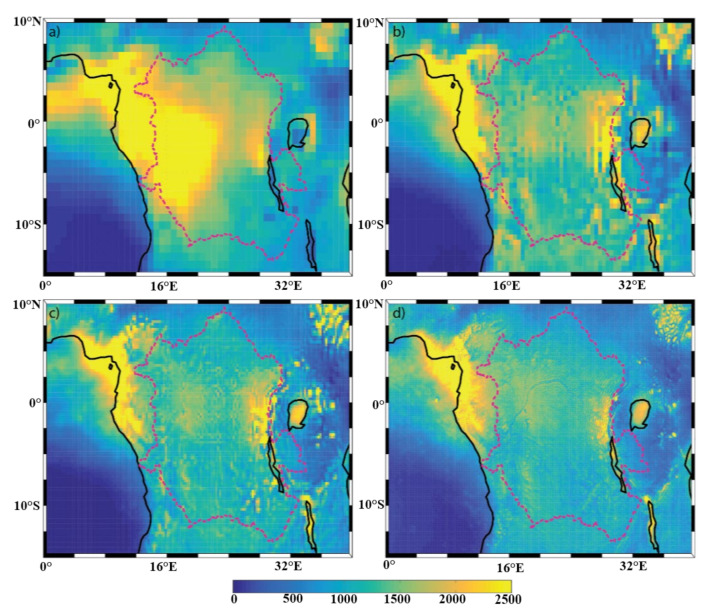
Spatial distribution of annual mean precipitation (mm/y) of the (**a**) ERA-I; (**b**) eERA5; (**c**) ERA5; and (**d**) ECOA datasets from 2016–2017. The Congo Basin is outlined with a dotted line.

**Figure 5:**
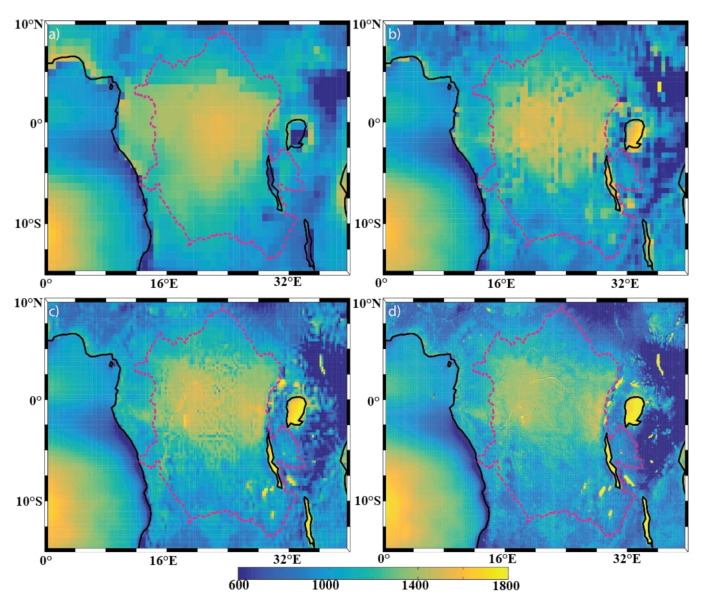
Spatial distribution of annual mean evaporation (mm/y) of the (**a**) ERA-I; (**b**) EERA5; (**c**) ERA5; and (**d**) ECOA datasets from 2016–2017. The Congo Basin is outlined with a dotted line.

**Figure 6:**
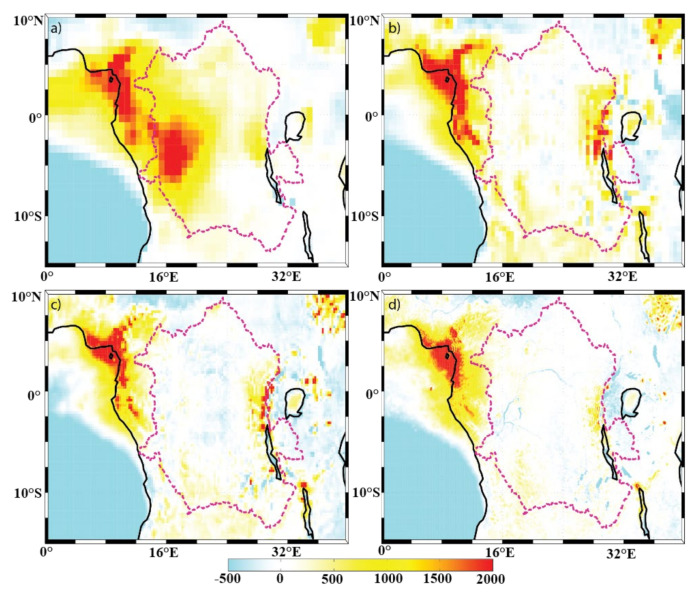
Spatial distribution of annual mean P-E (mm/y) of the (**a**) ERA-I; (**b**) eERA5; (**c**) ERA5; and (**d**) ECOA datasets from 2016–2017. The Congo Basin is outlined with a dotted line.

Figure 4, Figure 5 and Figure 6 show the mean annual precipitation (mm/y), evaporation (mm/y), and P-E (mm/y), respectively, for the four model resolutions over the Congo Basin and surrounding areas. It can be seen that ERA-I (Figure 4a) overestimates precipitation over the far western portion of the basin, but underestimates precipitation over the lakes located on the east side of the basin, as compared to the other datasets used (Figure 4b–d). The precipitation overestimation is a known issue within the ERA-I dataset [25,32]. Aside from the identified ERA-I precipitation biases over the western edge of the basin and over the lakes, the overall spatial patterns in annual precipitation generally agree across all resolutions. Examples of such precipitation features include the northwestern edge of the basin, the equatorial region, lakes Victoria, Tanganyika, and Malawi, and the noticeable minimum precipitation over the Great Rift Valley. Unsurprisingly, the datasets exhibit evident differences in the characterization of the finer-scale precipitation patterns. For instance, there are precipitation features found only in the ECOA dataset that are congruent with the topography of the Congo River, meaning that the Congo River is able to impact the precipitation field and that lower-resolution models will not be able capture these contributions.

The annual evaporation field (Figure 5) has a local maximum within the basin (in all 4 resolutions) along the equator. Evaporation from water surfaces (lakes) in ERA-I is typically weak, which is in contrast to the analysis from the other three datasets. This may be due to the impact of the land-surface scheme. The ERA-I IFS system used the Tiled ECMWF Scheme for Surface Exchanges over Land (TESSEL) land-surface scheme [40], while the hydrology TESSEL (HTESSEL) [50] was adopted in ERA5 and ECOA. Other resolution-independent features include imprints of the Great Rift Valley, the three lakes mentioned above, and the Atlantic Ocean evaporation minimum along 3° S where the Angola and Benguela ocean currents meet. As with the precipitation field, imprints of the Congo River can be observed only in the highest-resolution dataset ECOA evaporation field (Figure 5d).

The behavior of the moisture flux can be determined by comparing how the above-described features are represented in the P-E field (Figure 6). There is a local maximum P-E in all four resolutions along the northeastern coast, which coincides with the feature in the precipitation field. The ERA-I precipitation overestimation within the western sector of the Congo Basin (Figure 4a) is also apparent within the ERA-I P-E field (Figure 6a). The P-E is typically close to zero within the basin across the three higher-resolution datasets (Figure 6b–d), which suggests that there is local recycling of water within the basin on an annual basis. Imprints of the Congo River are also only visible in the 9-km P-E field. The P-E values over the lakes are also noticeably different between ERA-I and the higher-resolution datasets. The spatial patterns of the P-E over the Atlantic Ocean, however, coincide with those of the evaporation field.

Overall, the spatial patterns found in the mean P-E field are in fact a mixture between the precipitation and evaporation fields and are not overly dominated by either field. However, the comparisons between the mean states (climatology) cannot provide sufficient insight into the sources/contributions of the spatial variability in the P-E. The DCLS analysis is used to assess this.

### 3.2. Spatial Variability

**Figure 7:**
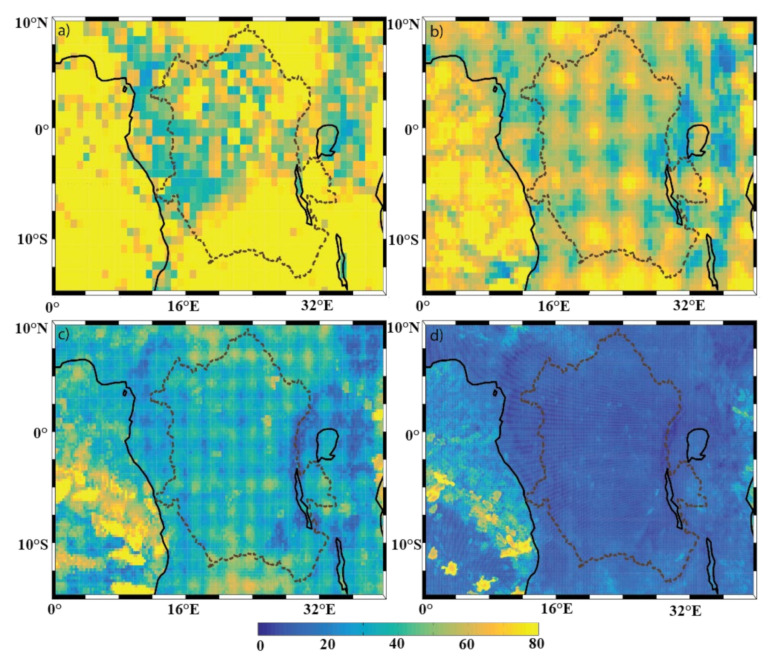
Spatial distribution of the precipitation decorrelation length scale (km) of the (**a**) ERA-I; (**b**) eERA5; (**c**) ERA5; and (**d**) ECOA datasets from 2016–2017. The Congo Basin is outlined with a dotted line. Panels (**a**) through (**c**) have a colormap ranging from 20–150 km, and panel (**d**) has a colormap ranging from 0–30 km. The color bar at the bottom is applicable to all panels.

**Figure 8:**
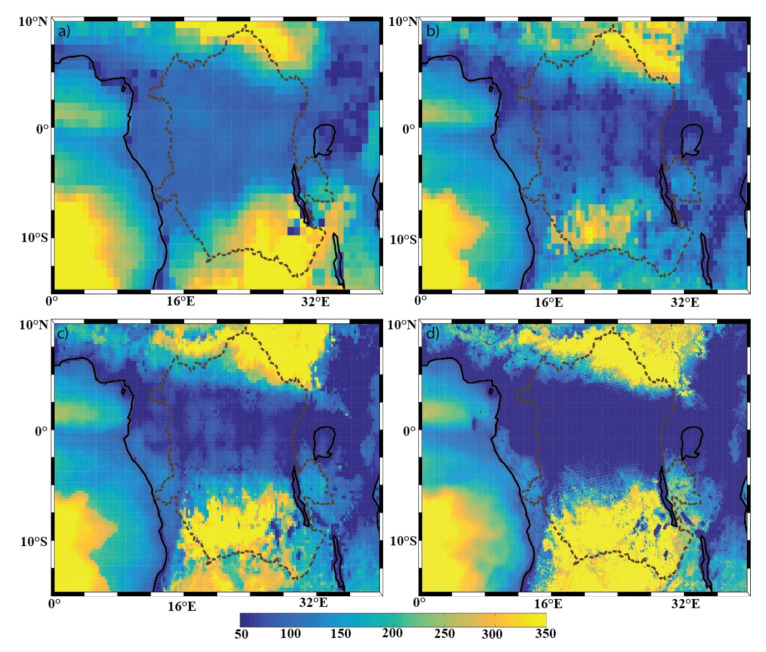
Spatial distribution of the evaporation decorrelation length scale (km) of the (**a**) ERA-I; (**b**) eERA5; (**c**) ERA5; and (**d**) ECOA datasets from 2016–2017. The Congo Basin is outlined with a dotted line.

The results of the DCLS analysis of the precipitation field are shown in Figure 7. Within a given resolution, the DCLS analysis is able to identify locations of relatively large spatial variability, i.e., local minima in DCLS. As explained in Section 2, whether a model resolution is sufficient or not can be determined by comparing the spatial patterns of the DCLS values from the current model dataset with those from higher-resolution datasets. Although there were some similarities in mean state across the four resolutions (Section 3.1), the spatial patterns in the DCLS values change drastically between ERA-I (Figure 7a) and ECOA (Figure 7d), indicating that the representation of the precipitation over the Congo Basin is bounded by the horizontal resolution of the model. This is not surprising since this region is characterized by convective precipitation systems, which usually exhibit strong spatial and temporal variations [27,31,32]. There is one spatial feature found in Figure 7b,d that should be noted, as it is likely a result of the background spectral model. There is, what looks like, checker boarding in the DCLS values within the central Basin. This is most likely Gibb’s phenomenon [51]. It is common for precipitation to have sharp changes in values (i.e., when it starts raining, the precipitation values abruptly change). Spectral models will represent values using a Fourier series—which have trouble modeling functions with sharp changes in values. The result of this difficulty can lead to small amplitude waves being propagated (sometimes referred to as a “ringing”). The Gibb’s phenomenon is also present in Figure 7d (the highest resolution at 9 km); however, at this resolution, the waves are clearly visible. Evidence to support the claim of this being Gibb’s phenomenon is that the evaporation field (Figure 8)—a field that does not exhibit the same volatility in values—does not exhibit this behavior.

The DCLS of the evaporation field (Figure 8), however, consists of both resolution-independent and dependent features. One resolution-independent feature is the equatorial minimum (which includes the minimum DCLS values within the basin), most likely as a result of the tropical rainbelt (also referred to as the Intertropical Convergence Zone, ITCZ). Furthermore, we see this minimum extend to the eastern region of the viewable domain. Although the spatial pattern does not appear to be distinct from the equatorial minimum, the high degree of spatial variability in this region is most likely explained by the topography of the Great Rift Valley (Figure 2). Another notable resolution-independent feature can be seen over the South Atlantic. There is a large amount of evaporation (Figure 5) but with a small spatial variability (i.e., large DCLS, Figure 8). Additionally, all datasets show the feature of small evaporation DCLS (high degree of spatial variability) along the coast, as well as over the 3° S latitude-ocean (where the different ocean currents meet). Given that the aforementioned DCLS features are not changed across different model resolutions, both the coarse-resolution and fine-resolution models are capable of capturing the large-scale spatial patterns of these features. It should be noted, however, that there is a subtle difference between a large-scale feature in the DCLS analysis and the associated amount of spatial variability. This does not mean that the model resolution is sufficient to capture variability within the identified large-scale pattern. For example, even ERA-I is able to capture the large-scale impact of the rain band; however, it appears that none of the resolutions are able to capture variability within the rain band.

In contrast, resolution-dependent DCLS features in the evaporation field mainly include the three lakes, shorelines, and any DCLS value that is less than 50 km in all four resolutions. The spatial patterns of the DCLS values were specifically checked for various color bar ranges. It was verified that there was no structure that was washed out by the chosen color bar ranges, thus there is no structure that occurs at <50 km that is washed out by this choice. It can be seen that there is an increase in fine-scale structure of the spatial patterns around the shorelines, including around the 3 lakes, at higher resolutions. Although the Great Rift Valley has a general minimum in DCLS that is apparent in all resolutions (as discussed above), subtle spatial patterns within the rift valley become increasingly noticeable with each increase in resolution. A similar phenomenon occurs with the equatorial minimum over the basin. Although there is a clear large-scale minimum in each resolution, we see some small differences in spatial patterns within the political perimeter (dotted line) of the basin. However, despite these small differences, there are still no signs of the Congo River in the 9-km ECOA resolution (Figure 8d)—a feature that the mean state was able to resolve at the 9-km resolution. One of the major reasons for these features is that the spatial variability of topography (Figure 2) and vegetation (Supplementary Figures S1 and S2) may not be sufficiently presented in coarse-resolution models. Lastly, the results of Figure 8 do not show imprints of vegetation, suggesting that the vegetation within the basin does not presently contribute to the spatial variability of the evaporation. Supplementary Figures S1 and S2 (percentage of high and low vegetation cover) show that there is nearly uniform vegetation coverage within the basin. Given that the vegetation field is spatially uniform, its impact on the evaporation field cannot be determined from this analysis. This means that this analysis is not able to determine how the evapotranspiration field will respond to changes in vegetation; however, this does not negatively impact the analysis of this study.

**Figure 9:**
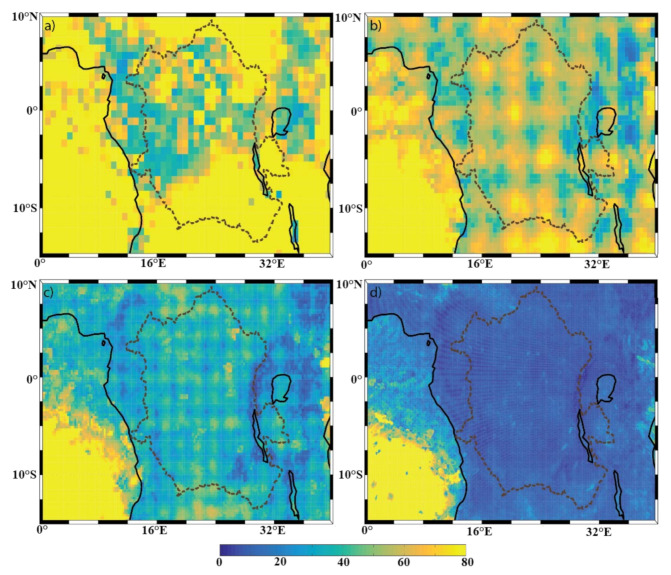
Spatial distribution of the P-E decorrelation length scale (km) of the (**a**) ERA-I; (**b**) eERA5; (**c**) ERA5; and (**d**) ECOA datasets from 2016–2017. The Congo Basin is outlined with a dotted line. Panels (**a**) through (**c**) have a colormap ranging from 20–150 km, and panel (**d**) has a colormap ranging from 0–30 km. The color bar at the bottom is applicable to all panels.

Now we compare the spatial variability of the individual precipitation and evaporation fields to the DCLS analysis of the P-E field (Figure 9). Similar to P, the P-E field features strong resolution-dependent variations in DCLS. Over the land and the equatorial Atlantic Ocean, the DCLS of the P-E field (Figure 9) closely resembles that of the precipitation field (Figure 7), illustrating that the spatial variability of the P-E field originates largely from the P field. In contrast, consistent with the DCLS analysis for the E field (Figure 8), P-E shows the resolution-independent DCLS maximum over the Southern Atlantic, where the annual evaporation is much higher than the annual precipitation (Figure 7).

As a reminder, the 2-year time series was a constraint of being the longest overlapping time period (at the time of analysis) between all 4 datasets. However, results from [42] show that a 2-year time series is sufficient and will not impact the validity of the results. To further support this claim, a sensitivity analysis was done using a 10-year and 39-year time series using ERA5—the second highest resolution dataset since ECOA has limited temporal coverage. The results of this sensitivity analysis can be seen in Supplementary Figure S5, showing the DCLS of the precipitation, evaporation, and P-E fields for each of three time windows (i.e., 1979–2017, 2008–2017, and 2016–2017). It can be seen that the spatial patterns between the three time windows are very similar and thus provide additional support that the results using the 2-year window are not negatively impacted by having a shorter time series; however, it has the added benefit of having a fourth resolution (ECOA).

### 3.3. Further Study of Choice of Contour Level for the DCLS Analysis

**Figure 10:**
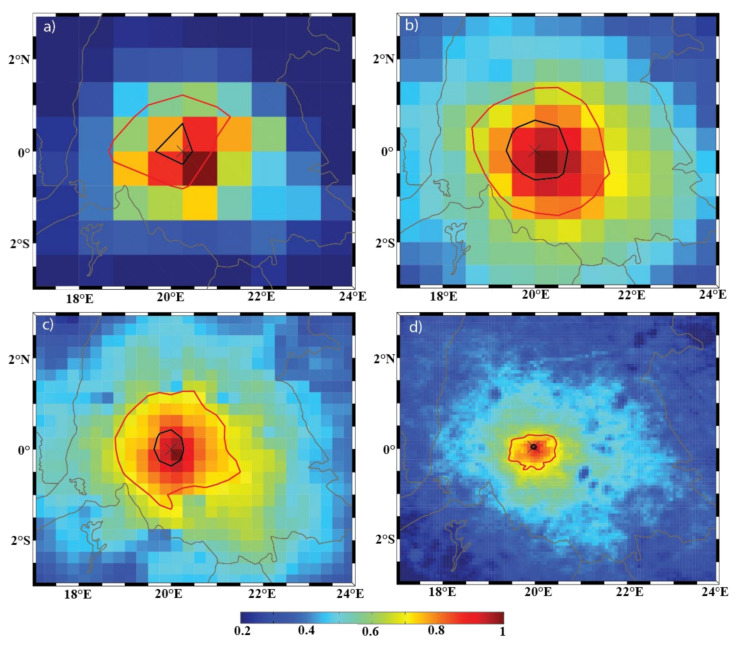
An example of the DCLS calculation of the precipitation field for the (**a**) ERA-I; (**b**) eERA5; (**c**) ERA5; and (**d**) ECOA datasets from 2016–2017. The background color represents the correlation coefficient values, the black contour represents the 0.9 contour level, and the red contour represents the 0.7 contour level.

As mentioned in Section 2, the magnitude of the DCLS values is known to depend on the choice contour threshold (correlation coefficient). A general trend is that the DCLS values will be smaller for higher contour thresholds, although the shift is not necessarily uniform. Figure 3 (which is also panel d in Supplementary Figure S7) can be used as motivation for this idea. The background coloring clearly resolves the spatial variability of the Congo River; however, a prescribed threshold of 0.7 would result in a contour larger than the river. In a case such as this, the corresponding DCLS value would not be much different over the river compared to over the surrounding land. Figure 10 shows an example of the 0.7 (red) and 0.9 (black) contour levels for the precipitation field at the location (20° E, 0° N). Since this is a similar analysis for the evaporation and P-E fields, the results of this contour analysis for these two fields are presented in Supplementary Figures S6 and S7.

Although Figure 10 shows some changes to the radius of the red 0.7 contour level across the four resolutions, the black 0.9 contour level shows a far larger change. The same pattern can be seen in the evaporation field (Supplementary Figure S6). The average radii of the 0.7 contour ranges from 240 km in ERA-I (Supplementary Figure S6a) to 153 km in ECOA (Supplementary Figure S6d), with a normalized standard deviation of 0.18 between the four resolutions. Conversely, the range in average radii for the 0.9 contour level is 24 (ECOA) km to 110 km (ERA-I), with a normalized standard deviation of 0.49 between the two resolutions. A similar pattern to that with the precipitation field (Figure 10) can be seen in the P-E field (Supplementary Figure S7)—which is in agreement with the results from Section 3.2—that the main source of spatial variability in the P-E field within this domain is the precipitation field. Although the DCLS values were smaller with a higher contour level (which is the predicable general trend), their sensitivities to model resolution differ. This result demonstrates the need to take care of the choice of contour level. In this study, the DCLS analysis was performed using both levels (0.7 and 0.9) to compare how the spatial patterns between the two results differed. If the choice of threshold does not affect the results, then the spatial patterns between the two analyses should coincide (with the only difference being the magnitude of the DCLS values). It was the case that the spatial patterns using the 0.9 contour level revealed more detail, which is why the results of the 0.9 contour threshold were presented in this study.

## 4. Discussion

The Congo Basin is one of the major river basins in the tropics. The atmospheric model datasets and satellite observations [52,53] play an important role in characterizing the basin’s hydro-climatological behavior since in situ hydrometeorological measurements are sparse in this region. However, it is not clear how the model resolution impacts the analysis and the understanding of the Congo Basin’s hydrology. Using the four sets of ECMWF’s IFS-based model (including reanalysis and operational analysis) data, we investigated the spatial variability pertaining to the precipitation (P), evaporation/evapotranspiration (E), and P-E fields in the region across different model resolutions (62 km, 31 km, 25 km, and 9 km) based upon the analysis of annual means and the DCLS technique.

As noted in the Introduction, the lack of in situ data has historically hindered scientific study in this region. This lack of data also makes it difficult to sufficiently validate models. Weather and climate models, due to the impact of model convective parameterizations, typically tend to overestimate the frequency of weak precipitation occurrences, i.e., the models produce precipitation more frequently and lightly than observed [54]. In ERA-I, an older version of the ECMWF’s IFS data assimilation system (IFS Cycle 31r2) was used, and less observations were assimilated. This is a likely cause of the precipitation overestimation over the Congo Basin in ERA-I, which is consistent with the findings from other studies [25,32,55]. Supplementary Figure S4 provides TAMSAT (version 3.0) satellite-based rainfall estimates [47,48,49] and their comparison with ECMWF’s reanalysis products over the Congo Basin. Overall, a reasonable agreement is found between TAMSAT and the four ECMWF’s products; however, here it is not practical to evaluate model data using in situ measurements over the Congo Basin due to the well-known scarcity of in situ observations.

In terms of annual means, all datasets feature the P and P-E maxima over the northwestern edge and the equatorial eastern edge of the basin and the E maximum over the equatorial basin. The lowest-resolution dataset (62-km ERA-I) also placed the extra P and P-E maxima within the western sector of the basin, while only the highest-resolution dataset (9-km ECOA) captured the features of the Congo River in these fields. The three higher-resolution datasets suggest that the mean annual P-E is close to 0 for most areas within the basin. However, the analysis of annual mean fields cannot sufficiently provide a quantitative estimation of the field’s spatial variability, which is examined by the DCLS analysis in this study. Results show that the analysis of P and P-E fields over the Congo Basin and the equatorial Atlantic Ocean are sensitive to the model resolution since the spatial patterns of their DCLS results are substantially changed across different model resolutions. A result of this is that the spatial variability of water fluxes in and around the Basin is not able to be fully characterized since the analysis suggests that the main structure of the variability occurs on scales less than 9 km. These results support the need for even higher resolution datasets where the DCLS analysis can be repeated to check for convergence in results. In contrast, the resolution-independent features are dominant in the DCLS analysis for the E field, indicating that the spatial variability of the E field mainly features large-scale patterns, which typically can be captured by both coarse-resolution and fine-resolution models. The comparison between the DCLS results from P-E and those from the individual P and E fields suggests that the P field is the dominant source responsible for the spatial variability of P-E occurring over land and the equatorial Atlantic Ocean, while over the Southern Atlantic, the P-E analysis is mainly governed by the E field, with both of them showing weak spatial variability.

The quantified impact of model resolution on the analysis of P-E over the Congo Basin could help characterize sources of uncertainty for hydro-climatological analyses in this region. For instance, Syed et al. [11] suggested that the Congo Basin (out of eight large basins) featured the least agreement between the observed discharge and P-E (as a proxy for discharge) derived from the NCEP-NCAR Global Reanalysis I (2.5° resolution) and ECMWF IFS CY25R1 operational forecast (spatial resolution of 55 km). Now a possible explanation is that the coarse model resolution for P-E impacted the performance of the P-E analysis.

The present study revealed a land–ocean contrast, as well as spatial variations in the response of P-E to model resolution. This means that the P-E analysis from the same model resolution typically performed differently at different locations. It is necessary to exercise caution when comparing the response of P-E to climate change over land and over the ocean [8] or the hydrological conditions between the Congo sub-basins [2]. The appropriate model-resolution datasets should be identified for different sectors.

Additionally, concerns regarding the impact of climate change and human activities on Congo Basin hydrology are increasing the development of hydrological models, especially high-resolution distributed models for this area. P-E (or P) is one of the major meteorological forcing inputs for driving hydrological models. The DCLS results from this study can provide important guidance for the selection of appropriate model resolution products as the hydrological model’s driver, thus mitigating the effects of uncertainties in P-E (or P) on the simulation analysis.

## Data Availability

The authors would like to thank the European Centre for Medium Range Weather Forecasts (ECMFW) for access to the ERA-I, eERA5, ERA5, and ECOA datasets. All data can be publicly and freely accessed through the ECMWF’s climate data store at https://climate.copernicus.eu/climate-data-store. The TAMSAT data used in this study is open access and were taken from https://www.tamsat.org.uk/data/archive (accessed on 20 November 2020).

## Supplementary Materials

The following are available online at https://www.mdpi.com/article/10.3390/s22010084/s1, Figure S1: Shows the amounts of high vegetation (as a percentage of area covered) for (**a**) ERA-I; (**b**) eERA5; (**c**) ERA5; and (**d**) ECOA. Figure S2: Shows the amounts of low vegetation (as a percentage of area covered) for **a**) ERA-I; (**b**) eERA5; (**c**) ERA5; and (**d**) ECOA. Figure S3: Comparison between DCLS values using a perscribed 0.7 contour and a 0.9 contour for the (**a**) precipitation field, (**b**) evaporation field, an (**c**) the P-E field for the ERA5 dataset over the domain shown in the other figures. Figure S4: Annual mean precipitation (mm/y) from TAMSAT-3 for 2016-2017. Below panel (**a**) are scatter plots comparing TAMSAT-3 precipitation to the (**b**) ERA-I, (**c**) eERA5, (**d**) ERA5, and (**e**) ECOA dataset using common grid points between each of the five datasets. In panels (**b** –**e**), the red line is the line of best fit, and the blue dotted line represents the 1-to-1 line. Figure S5: (**a**) The decorrelation length scale (km) of the precipitation (left column) and evaporation (right column) from the ERA5 over three different time spans to show covergence of results using only a tw-year window. (**b**) The decorrelation length scale (km) of the P-E field from the ERA5 over three different time spans to show covergence of results using only a tw-year window. Figure S6: An example of the DCLS calculation of evaportaion field for the the (**a**) ERA-I; (**b**) EERA5; (**c**) ERA5; and (**d**) ECOA datasets from 2016–2017. The background color represents the correlation coefficient values, and the black contour represents the 0.9 contour level and the red contour represents the 0.7 contour level. Figure S7: An example of the DCLS calculation of the precipitation field for the the (**a**) ERA-I; (**b**) EERA5; (**c**) ERA5; and (**d**) ECOA datasets from 2016-2017. The background color represents the correlation coefficient values, and the black contour represents the 0.9 contour level and the red contour represents the 0.7 contour level.
