# Supplementary material for: Representation of Spatial Variability of the Water Fluxes over the Congo Basin Region
Source: Sensors (Basel). 2021 Dec 23;22(1):84. doi: 10.3390/s22010084 (PMC8747179; doi:10.3390/s22010084)
Supplement: Supplementary file 1 [file sensors-22-00084-s001.zip › Supporting Information.pdf]

Supporting Information for

Representation of spatial variability of the moisture flux over the Congo Basin region

Marc De Benedetti[1,2]
G.W.K. Moore[1,2]
Xiaoyong Xu[2]
[1]*Department of Physics, University of Toronto*
[2]*Department of Chemical and Physical Sciences, University of Toronto Mississauga*

Table of Contents

i)	Figure S1	page 2
ii)	Figure S2	page 3
iii)	Figure S3	page 4
iv)	Figure S4	page 5
v)	Figure S5	page 6-7
vi)	Figure S6	page 8
vii)	Figure S7	page 9

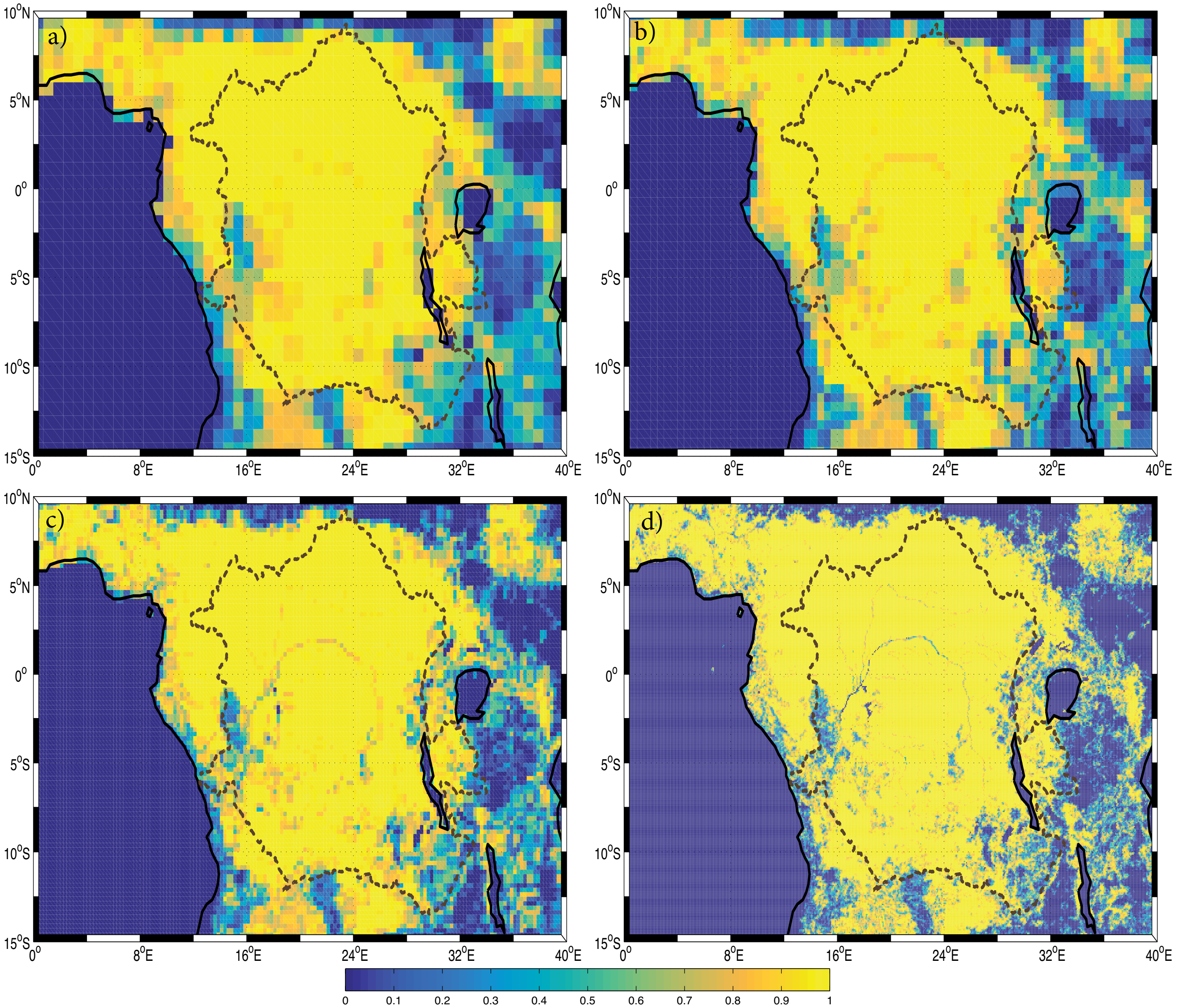

Figure S1: Shows the amounts of high vegetation (as a percentatge of area covered) for the a) ERA-I; b) EERA5; c) ERA5; and d) ECOA.

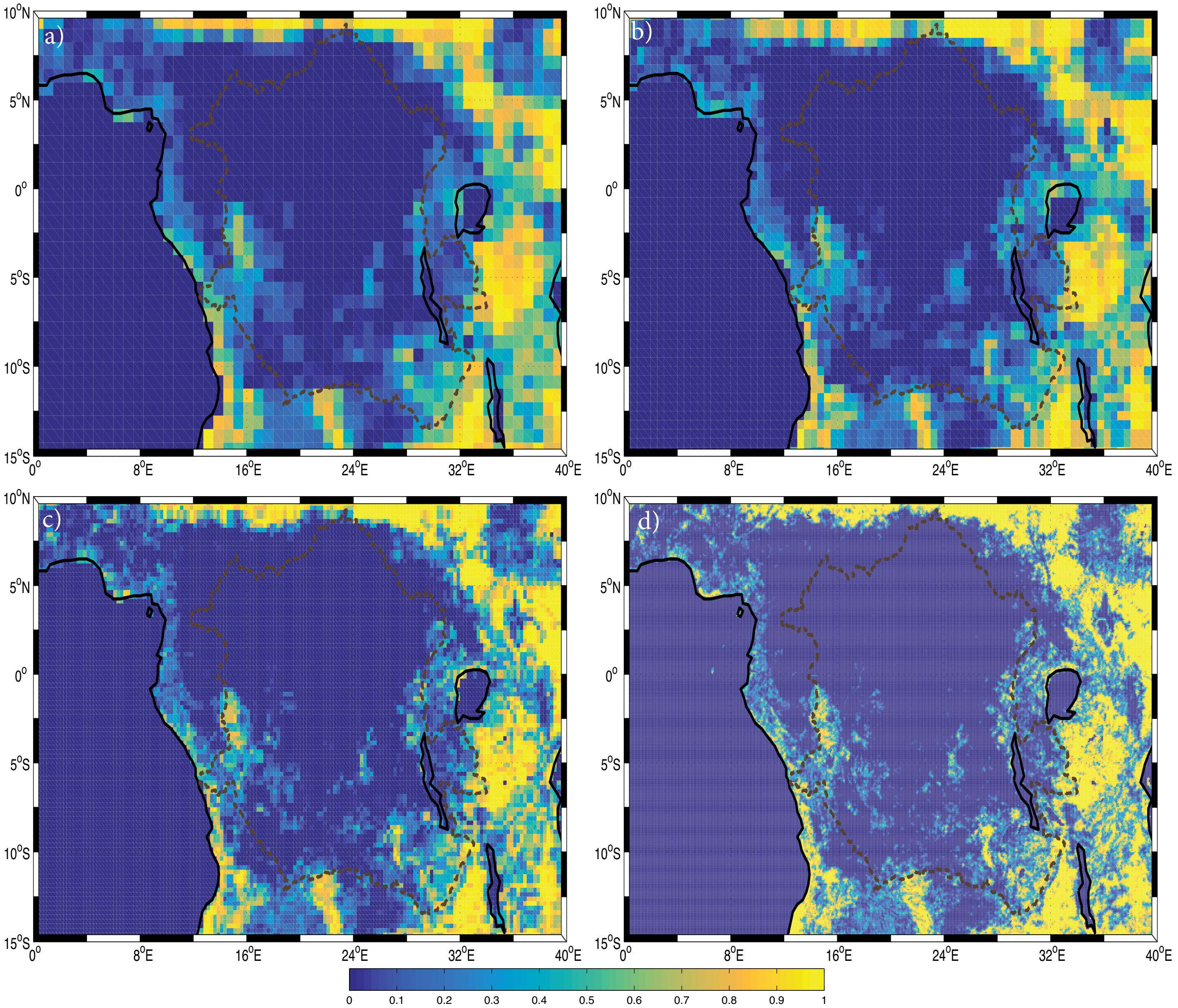

Figure S2: Shows the amounts of low vegetation (as a percentatge of area covered) for the a) ERA-I; b) EERA5; c) ERA5; and d) ECOA.

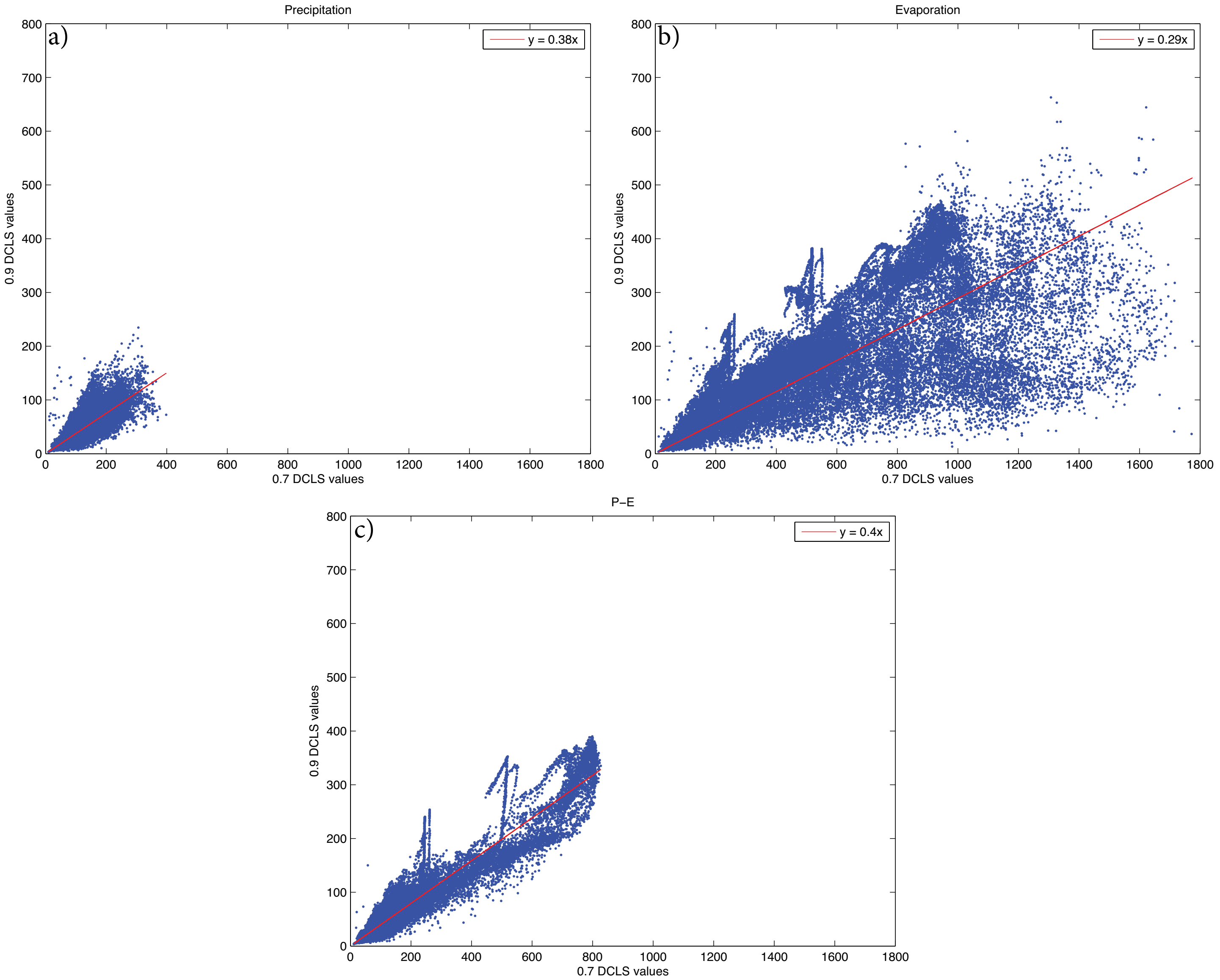

Figure S3: Comparison between DCLS values using a perscribed 0.7 contour and a 0.9 contour for the a) precipitation field, b) evaporation field, an c) the P-E field for the ERA5 dataset over the domain shown in the other figures.

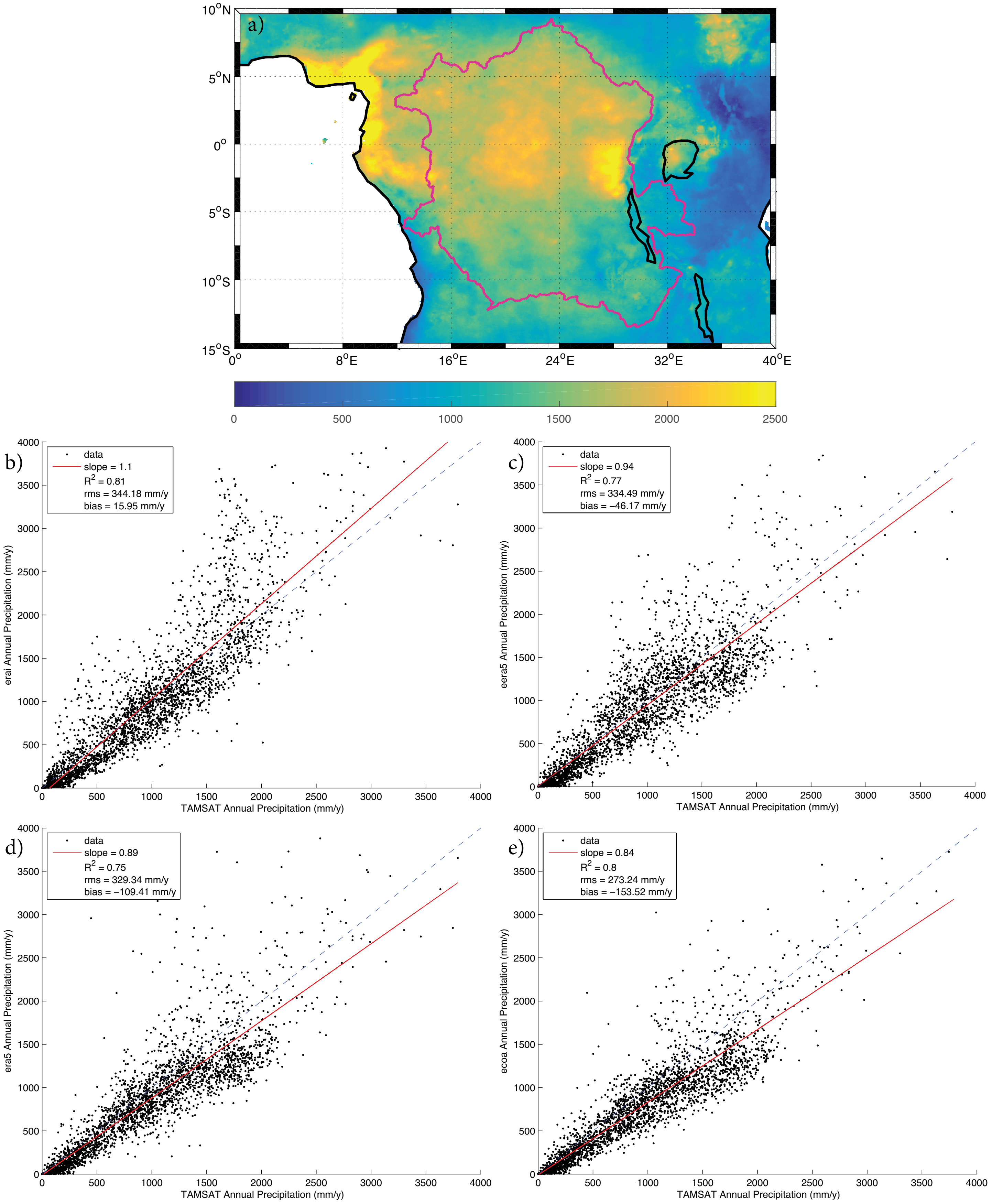

Figure S4: a) Annual mean precipitation (mm/y) from TAMSAT-3 for 2016-2017. Below panel a) are scatter plots comparing TAMSAT-3 precipitation to the b) ERA-I, c) eERA5, d) ERA5, and e) ECOA dataset using common grid points between each of the five datasets. In panels b) - e), the red line is the line of best fit, and the blue dotted line represents the 1-to-1 line.

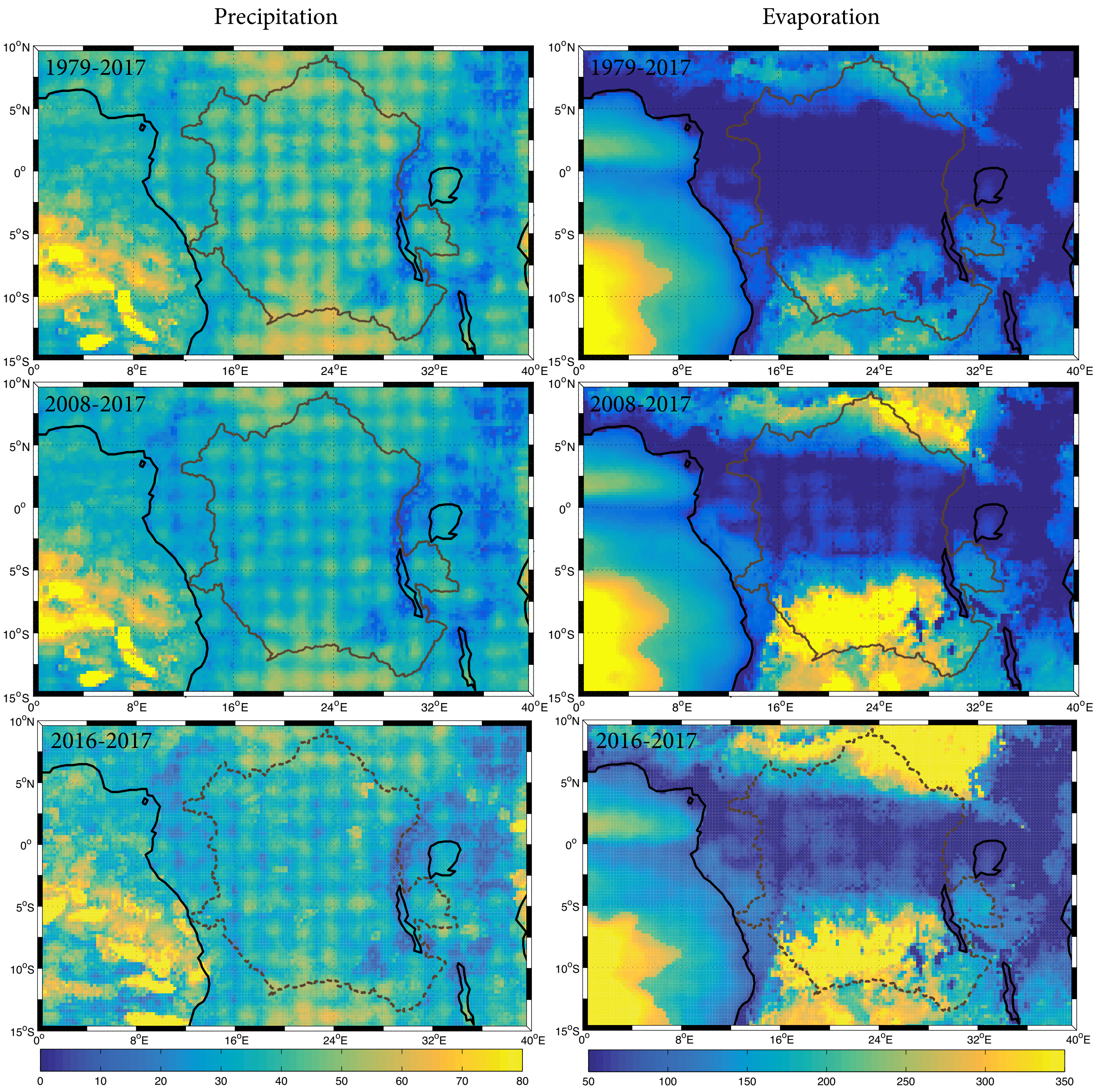

Figure S5a: The decorrelation length scale (km) of the precipitation (left column) and evaporation (right column) from the ERA5 over three different time spans to show covergence of results using only a tw-year window.

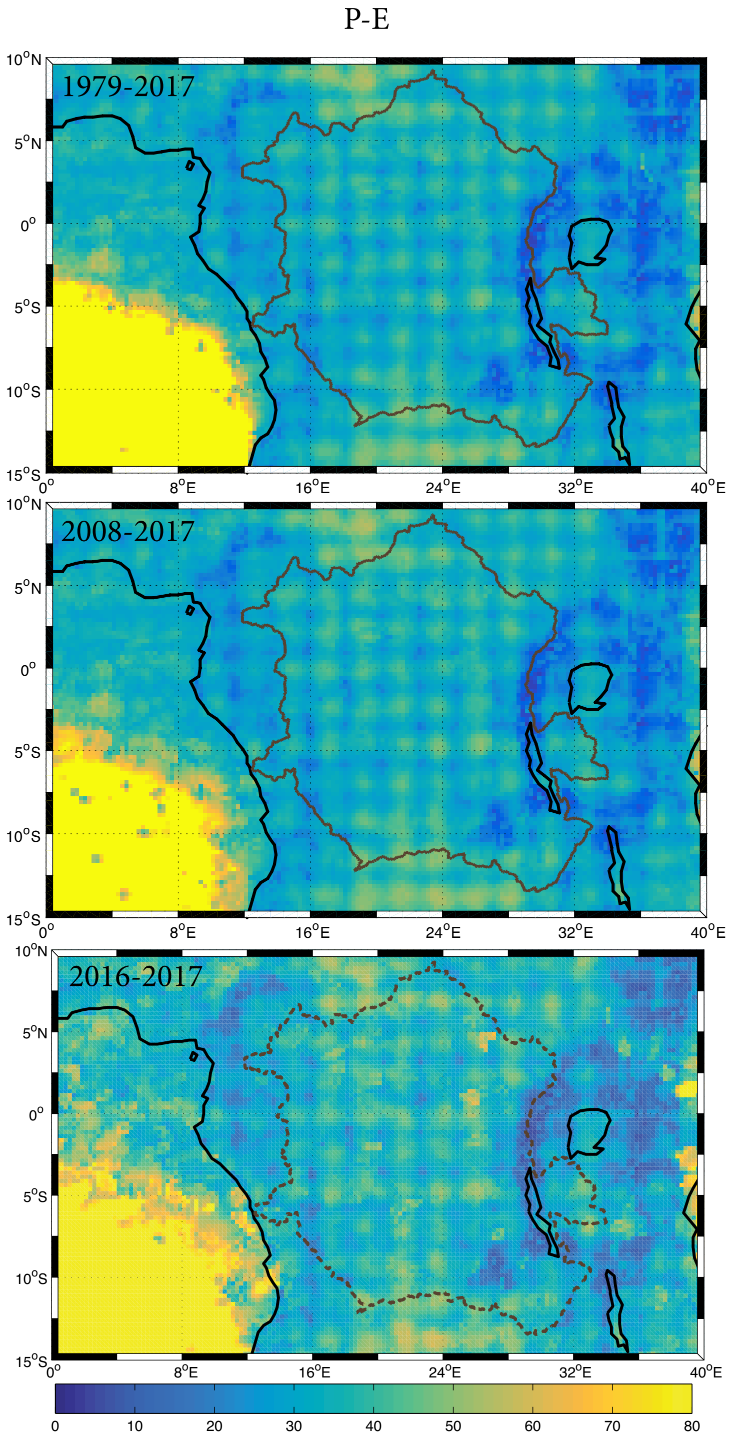

Figure S5b: The decorrelation length scale (km) of the P-E field from the ERA5 over three different time spans to show covergence of results using only a tw-year window.

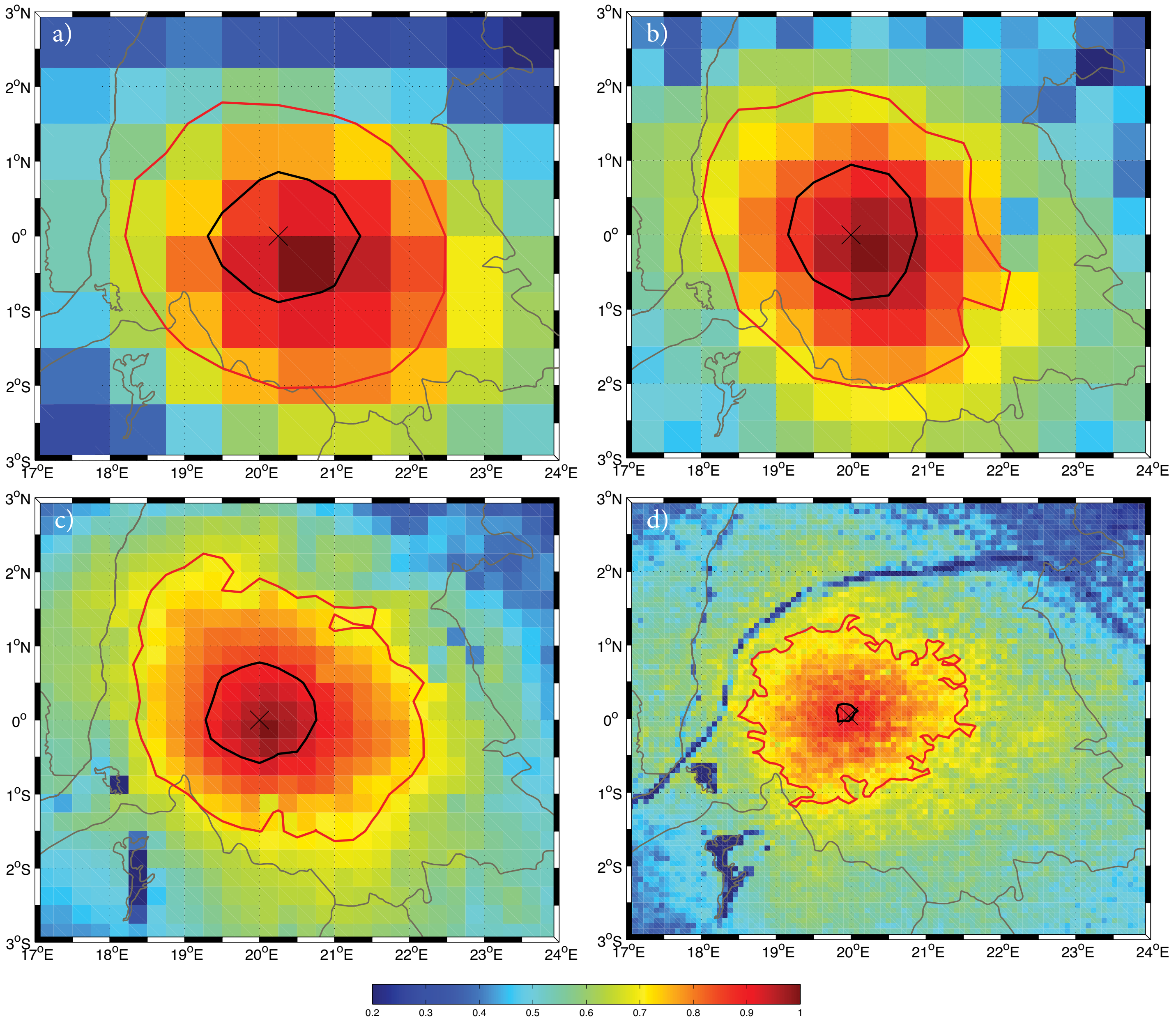

Figure S6: An example of the DCLS calculation of evaportaion field for the the a) ERA-I; b) EERA5; c) ERA5; and d) ECOA datasets from 2016-2017. The background color represents the correlation coefficient values, and the black contour represents the 0.9 contour level and the red contour represents the 0.7 contour level.

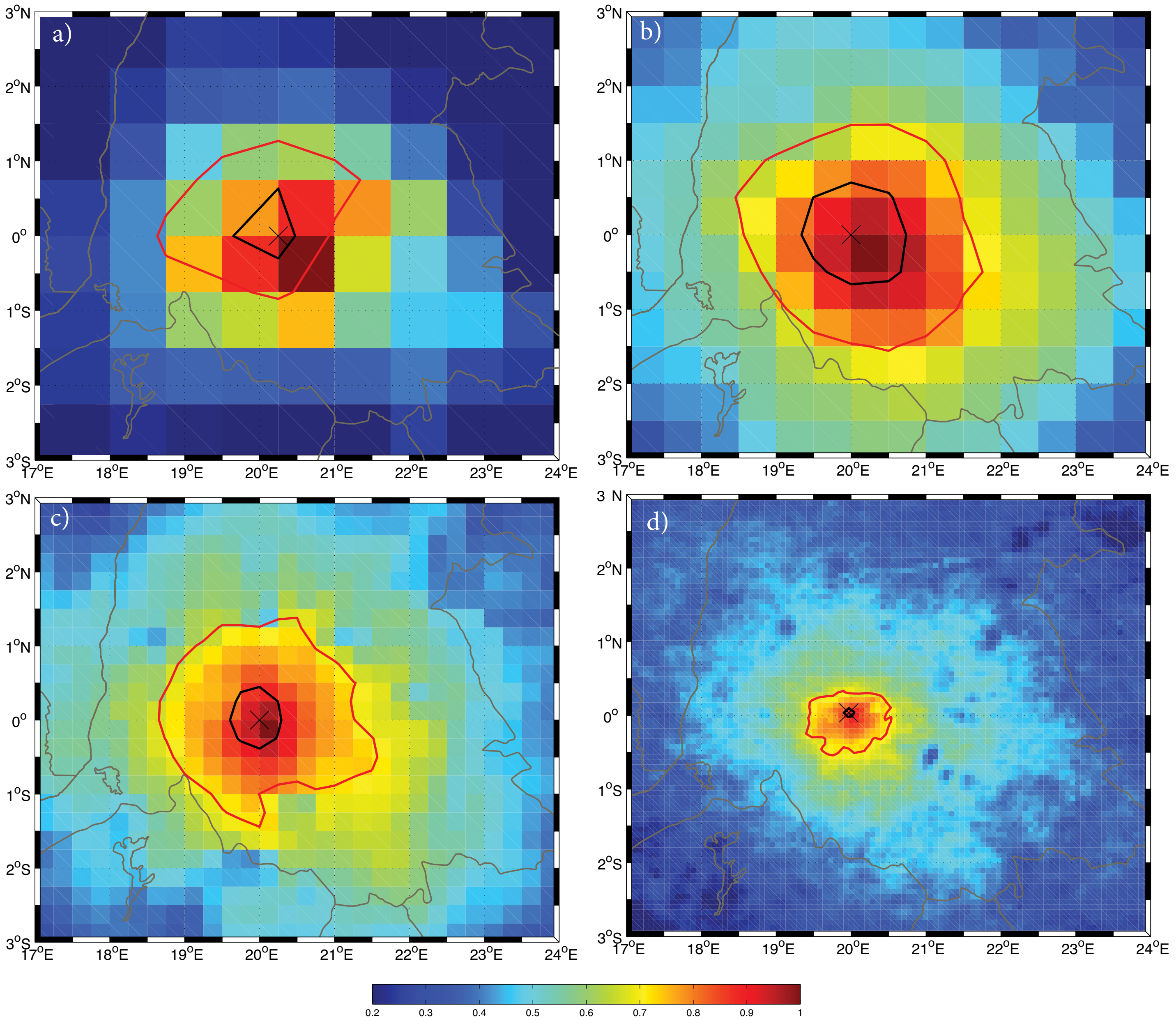

Figure S7: An example of the DCLS calculation of the precipitation field for the the a) ERA-I; b) EERA5; c) ERA5; and d) ECOA datasets from 2016-2017. The background color represents the correlation coefficient values, and the black contour represents the 0.9 contour level and the red contour represents the 0.7 contour level.
